# Etymologia: *Serratia marcescens*

**DOI:** 10.3201/eid2511.ET2511

**Published:** 2019-11

**Authors:** Gianluca Nazzaro

**Affiliations:** Foundation Istituto di Ricovero e Cura a Carattere Scientifico Ca’ Granda Ospedale Maggiore Policlinico, Milan, Italy

**Keywords:** Serratia marcescens, bacteria, nosocomial infections, urinary tract infections, enteric infections, prodigiosin, pigment, Serafino Serrati

## *Serratia marcescens* [sǝ-ra′-she-ǝ mar-ces′-cens]

*Serratia marcescens,* which can cause nosocomial outbreaks,and urinary tract and wound infections, is abundant in damp environments (Figure). It can be easily found in bathrooms, including shower corners and basins, where it appears as a pink–orange–red discoloration, due to the pigment known as prodigiosin. *Serratia* was discovered in Italy in 1819 when it affected polenta in a small town near Padua.

**Figure:**
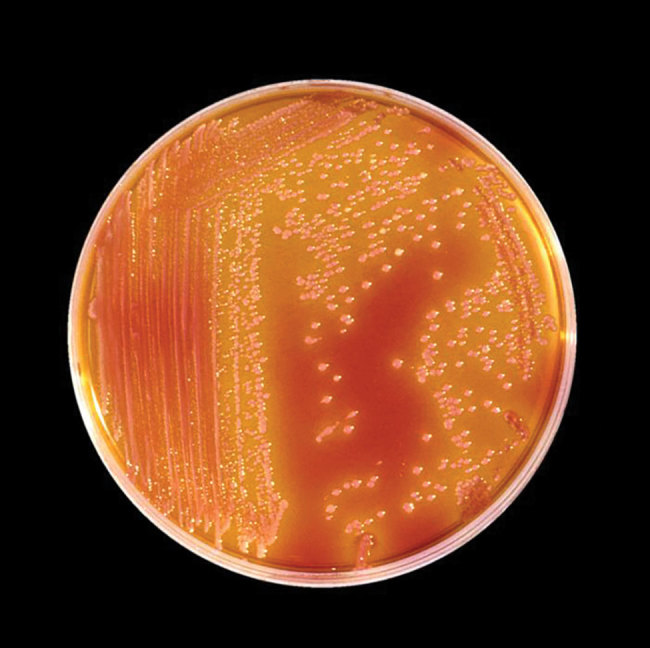
Culture plate containing the bacterium *Serratia marcescens*. The colonies are red because of a pigment (prodigiosin) produced by this organism. Source: Centers for Disease Control and Prevention, 1985.

Bartolomeo Bizio, a Venetian pharmacist, studied the mode of transmission of the red substance and named this microorganism *Serratia* in honor of Serafino Serrati, who ran the first steamboat on the Arno River in 1795, anticipating the discovery of Robert Fulton in 1807. The word *marcescens* was chosen from Latin for the species name meaning to decay, reflecting the rapid deterioration of the pigment. *Serratia marcescens* was later renamed *Monas prodigiosus* in 1846, then *Bacillus prodigiosus*, before the original name was restored in the 1920s in recognition of the work of Bizio. 
